# 

**Published:** 1861-12

**Authors:** 


### CONTENTS.---DECEMBER, 1861.

### ORIGINAL CONTRIBUTIONS.

PAGE

ART. I. OVARIAN TUMORS. By WM. H. BYFORD, A. M., M. D., Professor of Obstetrics and Diseases of Women and Children, in the Medical Department, Lind University, Chicago, Ill.,
1st Paper: –Is Ovariotomy a Justifiable Operation?.............................. 641
ART. II. The Therapeutical Uses of the Muriate of Ammonia. By M. G. HEYDOCK, M. D. 649
ART. III. Three Cases of Extirpation of the Eye. By E. L. HOLMES, M. D., ........... 655
ART. IV. A New mode of Counter Extension. By E. ANDREWS, A. M., M. D., etc.,.. 659
ART. V. Report on the Prevalent Diseases of the City of Chicago, for the last two Months. By N. S. DAVIS, M. D.,........................................ 661
ART. VI. Chloroform in Catalepsy. By ASHBEL WOODWARD, M. D., of Franklin, Conn., 666

### SELECTIONS.

Chlorate of Potassa—Some of its Uses. By Prof. S. R. PERCY,........................ 667
Surgical Notes from the *Lancet*,................................................ 670
Nitric Acid and Opium in Diarrhœa and Dysentery,................................ 673
Anæsthetics in Midwifery. By B. FORDYCE BARKER, M. D.,.......................... 674

### BOOK NOTICES.

Lectures on the Diseases of Women. By CHARDES WEST, M. D., Fellow of the Royal College of Surgeons, etc., etc.,.......................................... 677
Placenta Prævia; Its history and treatment. By WILLIAM READ, M. D., Member of the Mass. Medical Society,............................................... 681
The Gorilla: Being a Sketch of its History, Anatomy, General Appearance and Habits. By LEONARD J. SANFORD, M. D., New Haven, Conn., Dec., 1861, .................. 683
The Principles and Practice of Obstetrics. By GUNNING S. BEDFORD, A. M., M, D. Professor of Obstetrics, etc., etc.,.............................................. 685

### EDITORIAL.

*The Chicago Medical Examiner* for 1862,........................................ 686
Valuable Donation—Letter from Dr. LESCHER,....................................... 687
Army Sanitary Commission,......................................................... 689
Extracts from Correspondence—From Dr. S. W. WALLACE;—G. G. S., New York;—C. DUMREICHER, M. D., Wheeling, Va.,............................................ 690
Army Medical Society at Cairo,.................................................... 692
Work on New Remedies.—Obituary,................................................... 693
New Applications of the Fer-Chloride of Iron,..................................... 694
Physician's Hand Book, etc.,...................................................... 694
Mortality of Chicago—Mortality among Medical Journals—Clover Hay in Whooping-Cough—Personal,.......................................................... 695
SPECIAL SELECTIONS: Cure for Whooping-Cough—DUPUYTREN on Spurious Pregnancy—Large amount of Dropsical Fluid—Physical Training,............................. 696
Podophyllin as a Substitute for Mercury—Gun-Shot Wounds produced during the loading of Artillery,.................................................................. 697
Periosteal Reproduction of Bone,.................................................. 698
Reorganization of the Medical Department of the Army—Treatment of Child-Bed Fever,.. 699
Regeneration of Osseous Tissue,................................................... 700
Belladonna Shortening Labor,...................................................... 701
Puerperal Convulsions—Tarter Emetic in Asthma,.................................... 702

